# Moving the goalposts while scoring―the dilemma posed by new PET technologies

**DOI:** 10.1007/s00259-021-05403-2

**Published:** 2021-05-14

**Authors:** Julian M.M. Rogasch, Ronald Boellaard, Lucy Pike, Peter Borchmann, Peter Johnson, Jürgen Wolf, Sally F. Barrington, Carsten Kobe

**Affiliations:** 1 Department of Nuclear Medicine, Charité – Universitätsmedizin Berlin, corporate member of Freie Universität Berlin, Humboldt-Universität zu Berlin, and Berlin Institute of Health, Berlin, Germany; 2 Berlin Institute of Health (BIH), Berlin, Germany; 3 Radiology and Nuclear Medicine, Cancer Centre Amsterdam, Amsterdam UMC, De Boelelaan 1117, Amsterdam, The Netherlands; 4 King’s College London and Guy’s and St Thomas’ PET Centre, School of Biomedical Engineering and Imaging Sciences, King’s College London, King’s Health Partners, London, UK; 5 German Hodgkin Study Group, Department I of Internal Medicine, Center for Integrated Oncology Aachen Bonn Cologne Duesseldorf, University of Cologne, Cologne, Germany; 6 Cancer Research UK Centre, University of Southampton, Southampton, UK; 7 Department I of Internal Medicine, Center for Integrated Oncology Aachen Bonn Cologne Duesseldorf, University Hospital Cologne and University of Cologne, Cologne, Germany; 8 Department of Nuclear Medicine, University Hospital of Cologne, Kerpener Str. 62, 50937 Cologne, Germany

In this review, we provide a comprehensive description of the principles behind recent technological innovations, elucidate the current evidence, and point to specific fields of clinical care in which adoption of the most advanced technology, without careful thought, may put the previously established clinical role and benefits of PET at risk. In doing so, the authors will not argue that the introduction of new PET technology is in any way undesirable, but rather that during this process more emphasis should be placed on its ultimate effect on patient-relevant outcomes―on the possible limitations as well as the desired advantages.

This review belongs to a two-part series of reviews published in *EJNMMI* addressing the pros and cons of new PET technologies. The complementary review by Nicolas Aide et al. covers the pros [1].

## How should we evaluate the clinical benefits of PET and PET technologies?

The fundamental role of PET in clinical oncology is firmly established. This recognition of its clinical value has been achieved stepwise over time, by providing patients and clinicians with crucial additional information concerning tumor characterization, biology, and metastatic spread. However, this has been paralleled by concerns that in the average patient, PET may lead to overdiagnosis and overtreatment as a more sensitive imaging method. Before PET was introduced and before the concept of evidence-based medicine was widely adopted in the 1990s [2], there was generally no demand for a systematic and standardized assessment of the clinical benefit of any one imaging procedure. However, in evaluating the importance of PET imaging and PET technology today, medical societies and regulatory authorities ask for proof of actual clinical benefit to the individual patient―just as they do for development and approval of therapeutic drugs.

**Fig. 1:**
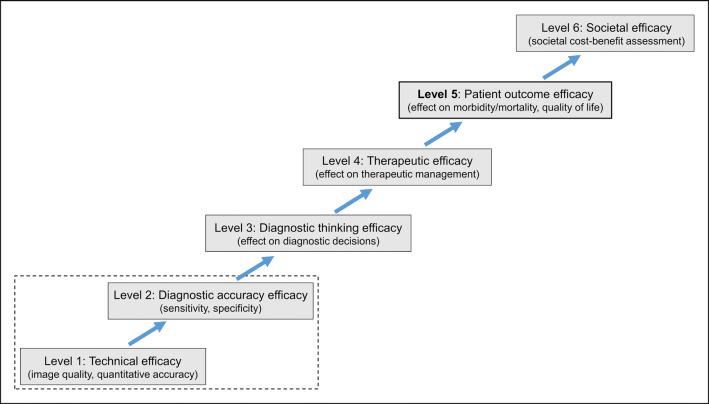
Levels of evidence in evaluating the efficacy of diagnostic imaging according to [3]. Please note that most studies on new PET technology only cover the first level or first two levels

Fryback and Thornbury have proposed six hierarchical levels of evidence for the clinical efficacy of diagnostic imaging (Fig. 1) [3]. In accordance with this system, for regulatory purposes, evidence of improved patient outcomes usually implies level 5 evidence. This requires proof of an advantage with regard to patient morbidity (e.g., as a consequence of a reduced rate of invasive procedures or futile surgeries), mortality, or quality of life [4]. In specific cases, proof of superior diagnostic accuracy may be sufficient if there is prior evidence from (randomized controlled) trials that the improvement in lesion/disease detection will lead to changes in treatment management that benefit patient outcome (e.g., early detection of disease relapse) [5]. However, such “linked evidence” is often difficult to establish, either because the study quality is too low or because there is no effective treatment to offer the patient [4, 6]. In the initial evaluation of a new test, patient-relevant endpoints may be replaced by surrogate endpoints if their direct relationship has been demonstrated based on biological plausibility and empirical evidence [7].

In a different scenario, use of a new diagnostic method can be justified if it has shown equal diagnostic accuracy in a well-designed study and provides other advantages over the standard method used [5] such as lower invasiveness, lower radiation exposure, or lower costs.

## Success stories in nuclear medicine: Introduction of PET in oncology

Prospective randomized controlled studies are regarded as the optimal method to demonstrate evidence for a clinical benefit by regulatory authorities. Accordingly, such studies have been major drivers in the past for the broad adoption of new radiopharmaceuticals and nuclear medicine techniques in routine clinical practice. However, as PET radiopharmaceuticals are often not patented, the perceived costs of patient scanning are high, and radiopharmaceutical availability may be limited, it remains especially difficult to find industrial sponsors willing to cover the costs of such expensive trials. Furthermore, not every clinical application of PET is equally susceptible to variability in imaging technology and requires highest-level evidence for its safe use. Nevertheless, there are fields in which seminal studies have been able to provide high levels of evidence for the additional value of PET, and selected studies are briefly outlined in the following paragraphs. The third section discusses the possible effects of “new” PET technology in these areas of successful application.

### PET in lung cancer

In a prospective, randomized controlled trial, [^18^F]fluorodeoxyglucose (FDG)-PET for staging patients with non-small cell lung cancer (NSCLC) revealed extensive mediastinal lymph node or distant metastases more frequently than conventional staging methods alone. Patients who did not undergo PET had progression or relapse within a year after surgery in 41% of cases as compared to 21% in patients who had undergone PET for staging. Thus, the addition of PET to the conventional workup prevented unnecessary surgery in one out of five patients (level 5 evidence) [8]. This study was confirmed by a randomized controlled trial by Fischer et al. [9] and led to the introduction of FDG-PET into standard protocols for the care of patients with NSCLC and potentially curative treatment [10, 11].

### PET in prostate cancer

[^68^Ga]Ga-prostate specific membrane antigen (PSMA)-11 was recently approved by the US Food and Drug Administration for PET in patients with prostate cancer [12]. This was based on two prospective studies showing that PSMA-11 PET has a high positive predictive value in assessment of lymph node metastases prior to surgery and in the detection of lesions in biochemical relapse. In either situation, this could have an important influence on therapeutic decisions (surrogate endpoint) [12, 13]. Hofman et al. recently reported a randomized controlled trial in 302 men with first diagnosis of prostate cancer before planned surgery or radiotherapy with curative intent. The authors showed that PSMA-11 PET/CT resulted in significantly more relevant changes in treatment management than conventional imaging (surrogate for level 5 evidence) [14].

### PET in lymphoma

FDG-PET has become the standard for staging and response assessment in patients with Hodgkin lymphoma (HL) and FDG-avid non-Hodgkin lymphoma. In the case of HL, the analysis of prospective, randomized controlled trials showed that bone marrow biopsy could be safely omitted if involvement had already been excluded by FDG-PET (level 5 evidence) [15–17]. In advanced stage HL, the prospective, randomized controlled HD15 trial demonstrated that radiotherapy can be restricted to 11% of patients after effective first-line chemotherapy based on the presence or absence of FDG-avid residual disease (level 5 evidence) [18]. Subsequent randomized controlled trials showed that a favorable PET result also allows a reduction of chemotherapy in advanced-stage HL [15, 19], may allow further de-escalation in selected patients in early stages of HL [20, 21], and can reliably guide selective administration of consolidative radiotherapy in advanced-stage diffuse large B-cell lymphoma (each level 5 evidence) [22].

### Summary

In the tumor entities and clinical settings described above, PET has been clearly shown to have a direct, beneficial influence on patient outcomes. In this way, nuclear medicine has succeeded in contributing to the treatment and cure of many oncological patients. However, the examples presented here have one important feature in common: the added benefit of PET imaging was primarily a consequence of its qualitative value, namely the functional information provided by the radiopharmaceutical and based on visual assessment, i.e., information on the presence of lesions, their location, and their spread (staging), not of its quantitative accuracy or precision or because of incremental improvements in image quality in an existing modality. It should be noted, however, that lack of imaging procedure guidelines and PET/CT system performance harmonization might have hampered the success of quantitative reads. Moreover, quantitative reads were mainly restricted to simple lesional uptake metrics, such as the SUVmax, while ignoring information on the location and spread of the disease. Radiomics analysis and quantitative dissemination features [23] can capture information on uptake variability (intra- and inter-lesional) as well as the spatial spread of the disease over the body and may thus provide a better and more clinically relevant quantitative surrogate for the disease aspects typically considered by visual inspection, as explained above.

## “New” PET technologies

### What is meant by “new” technologies?

New technologies for the purpose of this review comprise PET image reconstruction algorithms with point spread function (PSF) compensation and Bayesian penalized likelihood (PL) reconstruction algorithms as well as “digital PET” (i.e., using silicon photomultipliers (SiPM) instead of photomultiplier tubes (PMT)).

#### Point spread function reconstruction

The most common “conventional” PET reconstruction algorithm, ordered subset expectation maximization (OSEM), is iterative and involves a stepwise approximation of the tracer distribution that would have resulted in an image that is closest to the observed image. For this purpose, OSEM employed in PET imaging includes scatter and random correction, attenuation correction, normalization, dead time, and decay correction. This allows more accurate modeling of the observed distribution, especially in the low activity background [24].

PSF reconstruction algorithms are commonly based on the OSEM principle, but include an additional corrective term, which compensates for the scanner’s specific PSF. The latter describes the system’s depiction of point sources depending on their location in the field of view (FOV) [25, 26]. Due to the finite size of the detector elements, point sources at the periphery of the FOV are not perceived as a Gaussian activity distribution (as―ideally―in the FOV center) but as a skewed and degraded distribution curve. In non-PSF PET, this results in a steady decrease in spatial resolution from the FOV center to its periphery, which is compensated for by PSF reconstruction [26]. Notably, PSF reconstruction usually refers to the correction for F-18 while optimal PSF compensation for other radionuclides would require integrating their different PSF based on specific positron ranges [27, 28].

#### Bayesian penalized likelihood reconstruction

Standard OSEM reconstruction may not fully converge, i.e., the estimated probability of iterated images may never reach its maximum [29], especially as the iterative process is often stopped earlier (using a small number of iterations) to prevent excessive noise. An additional post-reconstruction filter is usually applied to achieve more visually appealing, smoothed images [30], which further counteracts the anticipated convergence of focal activity maxima.

PL reconstruction is also iterative, but the endpoint of iterations is determined by a penalization/regularization term instead of a fixed number of iterative steps. This term increases with increasing noise and regulates the overall function so that it reaches full convergence before developing excessive noise. Its relative strength is determined by a user-defined penalization/regularization factor β, which sets the tolerated level of noise in the final image (a higher β will result in lower noise level) [29, 31]. Another fundamental difference to OSEM is that currently applied PL reconstruction algorithms account for the relative differences between neighboring voxels, which enables a differentiated approach to be taken for voxels in the (ideally homogenous) background as compared to voxels in a “hot” or “cold” lesion [31, 32]. This aims at providing low background variability/noise while still achieving high contrast recovery (CR) of focal activity maxima. Since PL includes PSF compensation, the characteristics of this relative difference function regulate the amount of edge preservation (or smoothing) at sharp transitions between different activity levels. This balance is predefined by the developer [31–33]. PL reconstruction algorithms use the Bayesian principle by integrating estimates about the physical properties of the unknown image as a prior probability [34]. They can also be characterized as block sequential algorithms, as the sinogram is separated into blocks during the iterative process [29].

#### Silicon photomultipliers

The photomultiplier converts the photon emitted from the scintillation crystal into an electronic signal. The ideal photomultiplier would be of small size, provide full coverage of the scintillation crystal area (with no dead space between channels), be perfectly sensitive to single photons without the interference of dark current, and provide high single photon timing resolution <50 ps [35]. Compared to conventional photomultiplier tubes (PMT), SiPM in state-of-the-art PET/CT systems are smaller (enabling higher spatial resolution) and provide up to 100% coverage of the crystal area, as well as offering high sensitivity, low noise, and fast timing resolution [35]. PET/CT systems equipped with SiPM are commonly named “digital PET” although, strictly speaking, only SiPM with digital photon counting are fully digital [36]. Most current systems use analog SiPM, which generate a Geiger avalanche with a current signal that is proportional to the number of activated microcells [35, 37, 38]. Moreover, while all these current PET/CT systems use lutetium-based scintillation crystals, the SiPM design varies. This results in detector area coverage ranging from only 40% [39] to a full 100% [36, 40, 41], and a timing resolution ranging from 382 ps [42] down to 214 ps [40] as well as high ratios of SiPM channels per crystal element [39, 42] to the ideal 1-to-1 coupling of crystal to SiPM elements [36, 41].

### What was expected of these new technologies?

All three technological innovations aim at improving image quality (Table 1) and were introduced as game-changing technologies. The increase in image quality was expected to greatly increase diagnostic accuracy and giving clinicians and patients greater assurance that lesions would be detected with optimal quantitative accuracy [43–46]. Equally, the improvement in image quality (namely in noise properties) would, it was hoped, translate directly into a shortening of acquisition time by up to 90% [36, 47] or reduction in injected activity of up to 50% [36, 48–50]. Notably, this approach could be balanced in a way that can retain the accustomed image characteristics while achieving higher patient comfort or lower effective dose.

**Table 1:** Expected benefits associated with the new PET technologies

Feature	Superior diagnostic accuracy	Superior quantitative accuracy	Dose/time reduction
PSF reconstruction	+	(+)	−
PL reconstruction	+	+	+
SiPM	+	+	+

### Does the current evidence support new PET technologies?

#### PSF reconstruction

**Fig. 2:**
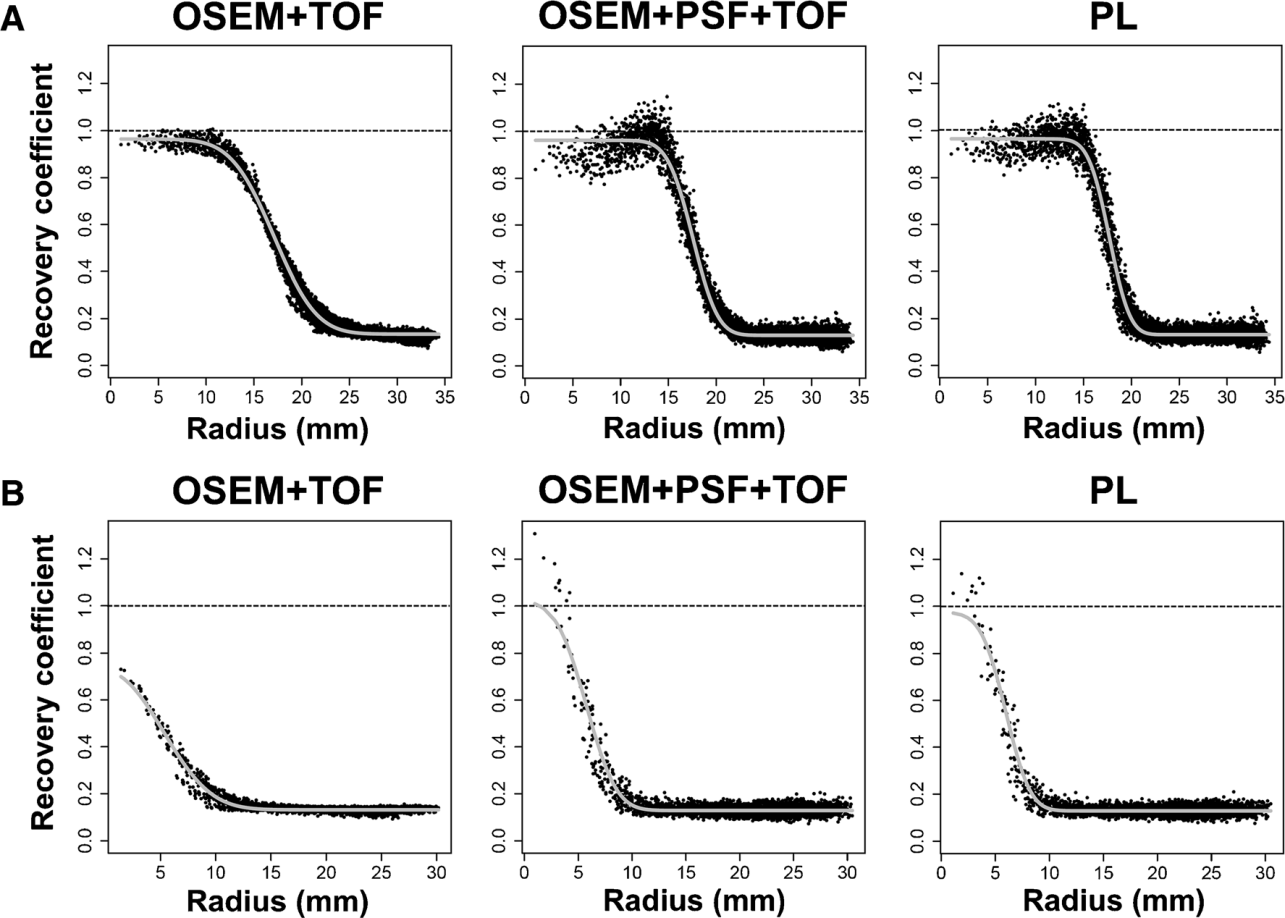
Radial activity profiles of “hot” spheres Radial activity profiles are displayed for “hot” spheres with a diameter of 37 mm (**A**) or 13 mm (**B**), respectively. These profiles are generated by arranging all voxel data (black dots) from the 3D image of the sphere on a 2D graph according to the voxel’s distance from the sphere center (center: “radius = 0 mm”). The true activity concentration in the sphere is represented by a recovery coefficient (RC) of 1.0 (dashed line). In the 37-mm sphere, OSEM accurately provides a maximum RC of 1.0, while PSF and PL reconstruction (β = 300) show overshoots and undershoots in the activity profile (edge artifacts) and overestimate the true activity concentration (maximum RC >1.0). In the small sphere, OSEM underestimates the activity concentration (maximum RC <1.0) while PSF and PL reconstruction (β = 300) again overestimate it. In clinical images, this would result in a higher SUVmax in small lesions with PSF and PL and potentially better lesion discernibility although this is a consequence of edge artifacts

PSF reconstruction improves the reconstructed spatial resolution [51, 52] and CR in small lesions [52–55]. However, it introduces so-called edge artifacts (edge elevations or Gibbs’ artifacts) that present as an overshoot (and undershoot) of activity at the sphere surface (illustration in Fig. 2 and [52]). Consequently, the true activity concentration can be overestimated over a range of different sphere sizes [52, 53, 56]. If sphere or lesion contrast is low, these edge artifacts are practically absent, but so is the potential improvement in reconstructed spatial resolution [52, 57]. To date, there is no consistent solution to these edge artifacts in conventional reconstruction algorithms, apart from applying a post-reconstruction filter to smooth the final data (and thereby negating the improved spatial resolution) [56, 58, 59].

Several studies have shown that the increasing lesion contrast improves lesion conspicuity/detectability (level 1 evidence; Table 2). However, lesion detectability does not necessarily imply improved sensitivity, specificity, or accuracy (see section “Effects on PET success stories”). Two studies about diagnostic accuracy in lymph node staging (level 2 evidence) have shown that PSF increases sensitivity while (slightly) reducing specificity [63, 64].

**Table 2:** Published studies on PSF reconstruction categorized by level of evidence [3]. Please note that this list is representative but not necessarily exhaustive. Furthermore, studies were included irrespective of positive or negative findings

PSF reconstruction	
Setting	Level of evidence
Phantom	Level 1: • Contrast recovery and SUV [52, 54, 55, 60] • Noise and image quality [61] • Reconstructed spatial resolution [51, 52] • Quantitative accuracy in microspheres [53] • Detection of simulated lesions [62]
Patients	Level 1: • Lesion SUV [57, 60, 63–67] • Image quality [61] • Image quality and lesion detection in PET/MRI [68] • Conspicuity of malignant lung lesions [69] • Lesion detection in prostate cancer biochemical relapse [70] Level 2: • Diagnostic accuracy in lymph node staging for lung cancer [63] • Diagnostic accuracy in lymph node staging for rectal cancer [64] Level 4: • Response assessment in lymphoma [71]

#### PL reconstruction

Several phantom and patient studies have shown that PL reconstruction can improve the trade-off between image noise and accurate CR of focal activity maxima compared to OSEM reconstruction. This could be interpreted as an improvement in overall image quality (level 1 evidence; Table 3). However, these studies also showed that the net benefit of PL reconstruction regarding image quality depends strongly on the β value chosen as well as the reconstruction parameters selected for the comparative conventional reconstruction algorithm [72]. A single study of level 2 evidence evaluated the diagnostic accuracy of OSEM with time of flight (TOF) compared to PL reconstruction, using a common β value of 400. In 121 histologically proven pulmonary lesions (either lung cancer, lung metastases, or benign lesions), diagnostic accuracy of visual assessment was unaffected by PL reconstruction in lesions ≤10 mm as well as >10 mm. Using an SUVmax cut-off optimized for each reconstruction, diagnostic accuracy was higher with PL reconstruction in lesions <10 mm. This was a result of increased sensitivity while specificity tended to decrease―a similar pattern as observed with PSF reconstruction [94].

**Table 3:** Published studies on PL reconstruction categorized by level of evidence [3]

PL reconstruction	
Setting	Level of evidence
Phantom	Level 1: • Contrast recovery [56, 72–75] • Contrast recovery in microspheres [76] • Noise [72–75] • Reconstructed spatial resolution [72]
Patients	Level 1: • Image noise [75–78] • Subjective image quality [73, 77, 79–84] • Prediction of subjective image quality from objectified measures [85] • Lesion SUV [75, 86, 87] • Conspicuity of pulmonary lesions [69, 77, 86] • Detection rate in pulmonary lesions [88] • Lesion detectability in pelvic PSMA PET/MRI at low activity [89] • Detection rate of lymph nodes in fluorocholine PET [90] • Normal databases for brain PET in neurodegenerative disease [91, 92] • Acquisition time reduction [81, 84, 89, 93] Level 2: • Diagnostic accuracy in pulmonary lesions [94] Level 4: • Response assessment in lymphoma [95]

Identification of a diagnostic benefit of PL reconstruction is further complicated by the requirement that an optimized β value must be obtained for different clinical scenarios and radiopharmaceuticals or activity levels [79–81, 86, 96]. To achieve a significant advantage in reconstructed spatial resolution and small lesion conspicuity may require β values as low as 150 [72, 86]. This will result in comparably high image noise, which may render the method inadequate for whole body imaging [72] unless PET systems with especially high sensitivity are used [42]. In clinical practice, β values between 300 and 600 have been recommended for whole body assessment with ^18^F- or ^68^Ga-labeled compounds [72, 73, 77, 79, 81–84].

Few patient studies have directly compared the image quality of PL reconstruction and OSEM-based reconstruction in order to evaluate their potential to reduce acquisition time. A potential to reduce acquisition time by 25 to >50% has been reported [81, 93]. Other authors have only investigated PL reconstruction at different β values [80, 84]. It is generally recognized that a reduction in acquisition time could equally well translate into reduced injected activity [97] and therefore into minimized radiation exposure. However, the studies cited here have mostly used noise level as a surrogate for image quality (evidence level 1). Reliable evidence of a secure reduction in acquisition time or activity would require proof of non-inferiority in diagnostic accuracy or other relevant clinical outcomes and, where relevant, non-inferiority in quantitative accuracy.

#### SiPM

Intra-individual comparison of SiPM and PMT in patients is complicated by the requirement to perform separate PET examinations as part of a prospective study, possibly combined with a higher radiation exposure due to a second CT scan. If both scans are performed using a single injection of activity, unbiased comparison implies a randomized scan order, because the later time point may be associated with systematically higher standardized uptake values (SUV), higher sensitivity yet higher image noise. Some previous studies have employed such randomized order, but no study beyond evidence level 1 has been performed in an unbiased protocol (Table 4). So far, no study has reported any possible disadvantages of SiPM technology regarding image quality or lesion detectability, which suggests that SiPM may constitute a systematic improvement over PMT. However, the actual clinical benefit of SiPM has not been studied. Further investigation is also required to assess whether there is a general, significant benefit associated with SiPM technology or whether this advantage is restricted to specific PET detector designs with high relative coverage of the detector area and especially favorable TOF characteristics [35, 38]. Zhang et al. estimated an increase in the effective sensitivity by a factor of 1.3 to 5.5 for activities of 7.4 to 337 MBq in a commercial SiPM PET system with a TOF resolution of 322 ps compared to a PMT system with comparable axial FOV length and 550 ps [47]. The anticipated further improvements in TOF resolution associated with SiPM [35] enable a further increase in effective sensitivity. The improved TOF performance of SiPM-based systems will likely also show benefit in image quality for patients with a regular weight (<90 kg) [105, 106]. This could be translated into an equivalent reduction in injected activity or acquisition time at equal image quality. Alternatively, the TOF gain could be exploited for an image quality or SNR gain equivalent to the square root of the relative improvement in timing resolution [105, 107].

**Table 4:** Published studies on SiPM categorized by level of evidence [3]

SiPM	
Setting	Level of evidence
Phantom	Level 1: • Spatial resolution and contrast recovery [42, 98–100] • Noise/background variability [42, 99, 100] • Lesion detection [101]
Patients	Level 1: • SUV in lesions and normal organs (randomized scan order) [102] • SUV in lesions and normal organs (non-randomized scan order) [96, 98, 103] • Lesion detection (randomized scan order) [49] • Lesion detection (non-randomized) [98, 103] • Acquisition time reduction (SiPM vs. PMT) [49] • Acquisition time reduction (SiPM only) [104] Level 3: • Staging (cTNM formula) in 5 patients with different tumors (non-randomized scan order) [103]

## Effects on PET success stories

In section ““New” PET technologies,” we discussed that the level of evidence for the clinical efficacy of new PET technologies is generally low, especially when compared to the body of evidence for the overall clinical value of PET imaging as outlined in section “Success stories in nuclear medicine: Introduction of PET in oncology.” In such specific clinical settings, the sought-for advantage of new technologies in lesion contrast and lesion detectability must be weighed against the risk of changing diagnostic and therapeutic strategies that have proven to be beneficial for patients, but where the evidence-base was established using established PET technology. The more susceptible the clinical consequence is to changes in image quality and lesion contrast, the more thorough the investigation of any potential undesired effects should be. Strictly speaking, the introduction of new methods and technologies would presuppose that they have shown non-inferiority―if not superiority―in the clinical endpoints routinely addressed by PET.

### Lung cancer

In patients with lung cancer, a possible advantage for patient outcomes could be established if new PET technologies helped to obviate invasive diagnostic procedures by improving differentiation between benign and malignant pulmonary lesions or by increasing the reliability of non-invasive lymph node staging. Furthermore, such imaging could benefit patients if futile surgery could be avoided through more accurate distinction of locally unresectable from resectable disease or through improved detection of otherwise unsuspected distant metastases.

However, there are currently no data on the effects of new PET technologies on lung cancer staging beyond level 2 (diagnostic accuracy). As described in section ““New” PET technologies,” PL reconstruction using a β of 400 did not improve diagnostic accuracy in the characterization of pulmonary lesions [94]. As reported by Lasnon et al., PSF reconstruction may improve sensitivity in lymph node staging; however, despite only a slight simultaneous increase in false positive findings, this could ultimately negate a potentially beneficial effect on lymph node staging [63].

The effect on patient-relevant outcomes remains equally unclear. In a recent single-center analysis [108], two PET systems were compared for pretherapeutic thoracic lymph node staging with FDG-PET/CT in patients with NSCLC, one scanner using PMT and OSEM with TOF reconstruction while the other was equipped with analog SiPM and PL reconstruction (β = 450). In addition to diagnostic accuracy per lymph node station, the potential to omit confirmatory invasive procedures (transbronchial biopsy) by modifying the diagnostic algorithm was investigated (surrogate endpoint for level 5 evidence). However, the analysis was retrospective, and the two scanners were used in separate patient cohorts. Therefore, neither intrapatient comparison nor randomization was available (although clinical key parameters were comparable in the two cohorts). Diagnostic accuracy of PET/CT per lymph node station was similar in the conventional scanner cohort (74.3%; *n* = 448 lymph node stations) and “digital” scanner cohort (79.8%; *n* = 252; chi-squared test, *p* = 0.1). More importantly, the frequency of invasive procedures that could have been avoided was similar in both cohorts (79.2% vs. 82.1%; *p* = 0.75) [108]. Consequently, in this context, SiPM and PL reconstruction did not appear to be superior. However, prospective intra-individual comparison or a randomized design would be required for a definite conclusion.

### Prostate cancer

In patients with prostate cancer, a higher sensitivity and diagnostic accuracy of PSMA-PET are eagerly anticipated considering its crucial role in detecting small PSMA-positive lesions in initial staging or in biochemical relapse. PSMA-guided treatment of single-lesion or oligometastatic relapse is being increasingly applied and may achieve favorable outcomes [109–112]. In this context, improved accuracy in detecting such lesions may result in superior progression-free survival after targeted treatment. In PSMA-PET, the relatively low injected activity can result in unfavorable noise levels, especially in overweight patients, which can complicate the detection of small lesions. Different groups have demonstrated an improvement in image quality for PSMA-PET using PL reconstruction (level 1 evidence) [81, 96, 113]. Alberts et al. recently compared a PMT-based and an analog SiPM-based scanner for [^68^Ga]Ga-PSMA-11 PET/CT in two matched retrospective cohorts with biochemical relapse of 88 patients each. Images from the conventional scanner were reconstructed with OSEM while the SiPM scanner used OSEM with PSF and TOF. Detection rates for both malignant (local recurrence, lymph nodes, bone) and benign lesions (e.g., ganglia or urinary tract activity) were about two times as high in the SiPM cohort compared to the PMT cohort. Pathological scans were significantly more frequent in the SiPM cohort compared to the conventional cohort in patients with prostate specific antigen (PSA) <2.0 ng/ml (level 1 evidence). Based on phantom measurements, the authors further showed that lesion contrasts and lesion discernibility were higher with the SiPM-based scanner especially in smaller lesions with 0.25 to 0.5 ml [98]. Such results constitute an important first step toward understanding the effect of new PET technology on patient care in prostate cancer and the emerging role of PET imaging in this context. However, as Fig. 1 illustrates, we are still a long way from demonstrating higher lesion counts as proof of a true benefit to patient outcomes.

### Lymphoma

The use of FDG-PET in lymphoma has brought the highest-level evidence of its benefit to patients. The unique feature of PET imaging in these patients is that it provides a more reliable assessment of chemosensitivity during treatment and residual viable disease after treatment (especially after induction chemotherapy) than clinical criteria and CT. More reliable assessment allows adoption of a tailored, less intensive, and less toxic treatment for the majority of patients who respond to therapy. Demonstrating this direct connection between the occasionally subtle PET findings and fundamental treatment decisions has required laborious and large-scale prospective studies as well as the development and acceptance of common criteria for PET interpretation.

Between 2003 and 2008, the HD15 trial for advanced stage HL showed that radiotherapy after effective chemotherapy can be restricted to the 11% of patients who showed PET positive residual lesions [18]. The cut-off used for PET positivity was the uptake in mediastinal blood pool structures (“Deauville score 3”) [114]. In the subsequent HD18 trial for advanced stages starting in 2010, the restriction of radiotherapy to PET positive residua was the new standard carried over from HD15. Unexpectedly, the proportion of patients showing PET positive residua and consequently undergoing radiotherapy increased from 11% in HD15 to 30% in HD18. What went wrong? In HD15, mostly stand-alone PET scanners were used, whereas by the time of HD18, technology had improved, and combined PET/CT scanners had become the standard. The practical solution for keeping the rate of patients undergoing radiotherapy close to 11% was to switch the visual cut-off for PET positivity to uptake higher than that of the liver (“Deauville score 4”) [15].

Later, Barrington et al. also reported a shift toward more positive reads for PET studies with subsequent clinical consequences [115]. This shift was found to coincide with the introduction of a new generation of PET/CT systems with PSF reconstruction. A systematic increase in Deauville scores can be expected as PSF increases the uptake, especially in small lesions [53, 57] such as residual tumor tissue but not in large, homogenous organs such as the liver and blood pool [60]. Reliable use of the well-established Deauville score could thereby be hampered. Enilorac et al. [71] compared PET scans in diffuse large B-cell lymphoma reconstructed with PSF by applying either no post-reconstruction filter or a Gaussian filter that complied with the EANM Research Ltd. (EARL) harmonization standard [116, 117]. The authors concluded that the influence of PSF on imaging results was limited, because major discordances (PET positive vs. negative) at interim staging affected only 5% of all cases. However, focusing on cases with Deauville score 3 based on the EARL-compliant reconstruction, 4 of these 22 patients (18%) with residual tissue were given a divergent rating of Deauville score 4 using the unfiltered PSF reconstruction [71]. Similarly, 3 of 18 patients (17%) with Deauville score 3 were upgraded to Deauville score 4 by unfiltered PSF reconstruction in end-of-treatment scans [118]. Ly et al. used the PET data from 52 patients with lymphoma to compare PL reconstruction (β = 500; compliant with the updated EARL specifications [56, 119]) with OSEM (without PSF or TOF; compliant with the first EARL standard). PL reconstruction led to a Deauville score 4 in 4 of 31 patients (13%; scoring based on the SUVmax) or 4 of 30 patients (13%; SUVpeak) who were rated as Deauville score 3 with OSEM [95]. In both the above studies, these observations were of immediate clinical importance because the cutoff between Deauville scores 3 and 4 is currently used to distinguish responders from non-responders. To show that such changes do not alter the progression-free and overall survival, large trials with sufficiently long follow-up will be needed to provide sufficient statistical power.

## Summary and outlook

In this review, we have argued that a true advance in imaging technology should eventually be shown to improve patient outcome or other measures of patient benefit and that this is yet to be demonstrated for the latest PET technologies. If they introduce variability and decrease standardization, as illustrated for the Deauville score, without a net improvement in clinical outcome, there are good grounds for remaining cautious about their role in clinical care.

Comparability of image characteristics and quantitative results is a general issue in medical imaging, and is not confined to PET imaging, let alone to the “new” technologies. Unfortunately, SUV differences between different scanner generations and reconstruction algorithms cannot simply be eliminated by normalization to a reference region such as the liver [60, 102]. Retrospective harmonization of quantitative reads may be achieved by a data transformation method, called ComBat [120]. This method uses the quantitative reads within a multicenter setting to derive a data-driven transformation and aligns the distribution of data values among centers. This method may be useful when developing or validating evaluation criteria when data is collected in a non-harmonized fashion. However, due to the nature of data-driven transformations, the method needs to be trained for each specific combination of multicenter sites, PET/CT systems, and for each dataset (i.e., disease type and stage). Therefore, the obtained transformation cannot be directly or prospectively applied to other multicenter datasets, diseases, and stages nor can it be derived from phantom studies. Furthermore, covariates need to be considered carefully [120].

**Fig. 3:**
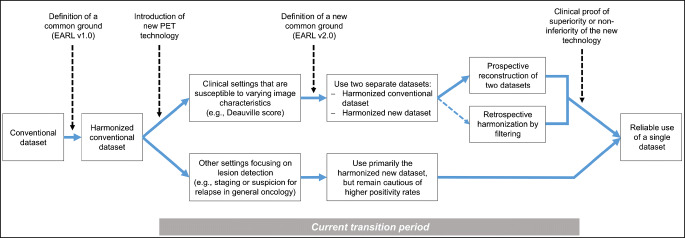
The long but safe route to adoption of new PET technology in routine clinical practice

Fortunately, the nuclear medicine community has been eager to characterize and address this variability by introducing PET/CT performance standards aiming at realizing upfront and generalizable harmonization. For conventional scanners with OSEM-based reconstruction, the first EARL standard ensured that all systems achieved sphere recovery coefficients (RC) within a common range [116, 117]. Following the same approach, Kaalep et al. have demonstrated that a new standard can be defined based on a different range of sphere RC that is now satisfied by PET/CT scanners with PSF or PL reconstruction, some of them equipped with SiPM [56]. Currently, the safest way of applying new technologies in clinical routine practice is to have two distinct datasets available―one that is compliant with the conventional standard and one that fulfills the new standard at least until their effects on image interpretation and subsequent patient management are better understood (Fig. 3). Ideally, these datasets should be reconstructed prospectively. As an alternative, retrospective smoothing of the new datasets, usually with a Gaussian filter of specific width, can be employed to achieve image characteristics that are comparable to the conventional standard [58, 59, 119]. It may be noted that the EARL standards were introduced to harmonize data for visual assessment and SUV calculations in oncological whole-body F-18 PET with the goal of achieving sphere CR at high contrast that are comparable within a range of about ±10% [56]. However, different clinical tasks, target lesion characteristics, or radionuclides will warrant validation of the methodology and―potentially―different or additional steps to achieve the necessary level of harmonization. As an example, a much smaller range of CR of about ±3% between state-of-the-art PET scanners has recently been demonstrated in F-18 studies using a Hoffman 3D brain phantom. This required task-specific quantitative measures, reconstruction settings, and image-based normalization [121]. Regarding radiomics features, Pfaehler et al. showed using patient-derived 3D prints that considerable variability in features remained between different PET scanners―despite the improved agreement achieved with EARL-compliant reconstruction [122]. This suggests that such applications may warrant additional efforts, such as the named ComBat approach [120]. EARL-compliant reconstruction has been successfully transferred from F-18 to Ga-68 and Zr-89. However, thorough correction for radionuclide-specific cross-calibration mismatch was necessary to ensure compliance [123, 124].

If an appropriate new standard has been established, the next step is to characterize the implications of this new standard for clinical decisions in appropriately designed clinical trials for specific clinical scenarios. Apart from laborious randomized trials, this might also be done as post hoc analyses such as those performed in HD17 and HD18 to explore thresholds that discriminate response better using the different technologies/datasets. This would include determining the degree to which the established and the new dataset differ―especially for those patients where uptake is close to the decision threshold―and evaluating the potential for management change. New metrics that may be more suited for advanced reconstructions, e.g., based on SUVpeak for lesional uptake [56, 72], should also be explored in such clinical trial datasets. Besides Deauville scores and other established diagnostic cut-offs [65], such studies may address the influence on prognostic cut-offs [58] or on normal databases [91, 92]. Using either approach, non-inferiority of the new standard regarding clinical endpoints must be well proven, before it can be used safely to guide crucial clinical decisions. Notably, post hoc analyses may be of limited validity as the effect of using the alternative, new dataset for therapeutic decisions regarding patient outcome may only be assessed indirectly―if at all.

Recently there has been interest in use of virtual (or in silico) imaging trials whereby the patients, imaging system, and clinical interpretation processes are emulated using computational models [125–127]. If properly validated, virtual imaging trials could have a role in evaluating the impact of new technologies on diagnostic and quantitative accuracy, thus decreasing the required size and length of real clinical trials and reducing the length of the transition period.

## Declarations

This article does not contain any studies with human participants or animals performed by any of the authors.
